# Off-Duty Internet Use and Depression: The Japanese Civil Servants Study

**DOI:** 10.31662/jmaj.2025-0505

**Published:** 2026-07-10

**Authors:** Daiki Fukushima, Takashi Tatsuse, Michikazu Sekine, Masaaki Yamada

**Affiliations:** 1 Faculty of Medicine, University of Toyama, Toyama, Japan; 2 Department of Epidemiology and Health Policy, University of Toyama, Toyama, Japan

**Keywords:** civil servants, depression, internet use, job stress, mental health, work-family conflict

## Abstract

**Introduction:**

Both on-duty and off-duty internet use are increasing and may have health effects. Off-duty internet use, in particular, has increased rapidly due to the proliferation of smartphones. This study aimed to clarify the relationship between prolonged off-duty internet use and depression among Japanese civil servants.

**Methods:**

A cross-sectional survey was conducted in 2019 among 3,751 civil servants aged 20-65 years employed by a local government on the west coast of Japan. Psychosocial stress at work, work-family conflict, and time spent using the internet were evaluated. Logistic regression analysis was performed to examine whether there is an association between off-duty internet use time and depression as measured using the Japanese version of the Center for Epidemiologic Studies Depression Scale.

**Results:**

The study included 3,403 participants; depressive symptoms were present in 28.3% (26.1% of men and 31.4% of women). Multivariate analysis showed that, in women, prolonged off-duty internet use, low job control, low job support, being unmarried, work-family conflict, and low sleep quality were associated with depression. In men, in addition to these factors, low job class, no overtime work, high job demands, chronic illness, and sleep duration were associated with depression. While multivariate analysis revealed no statistically significant association between electronic device use at work and depression, prolonged off-duty internet use (≥4 hours per day) was significantly associated with higher odds of depression in both men (odds ratio [OR] 1.83, 95% confidence interval [CI] 1.08-3.10) and women (OR 2.31, 95% CI 1.32-4.05).

**Conclusions:**

Prolonged off-duty internet use was independently associated with depression among Japanese civil servants. This association may be related to factors such as superficial virtual connections or information overload. More attention should be paid to internet use for maintaining the mental health of civil servants.

## Introduction

The World Health Organization reported that 15% of the global working-age population experienced mental disorders in 2019 ^(1)^. It is estimated that 12 billion working days are lost annually owing to depression and anxiety disorders, with estimated annual productivity losses reaching US $1 trillion. Worker mental health is, therefore, a significant public health concern and an economic issue.

The incidence of stress-related illnesses is increasing in Japan. In 2022, the Ministry of Health, Labour and Welfare reported that the proportion of workplaces with employees absent for >1 month or who resigned due to mental health issues over the past year was 13.3%, an increase of 3.2% compared to the previous year ^(2)^. Additionally, 82.2% of the workers indicated that their work or professional life caused anxiety, worry, or stress, up notably from 53.3% the previous year. These survey results underscore a significant prevalence of mental health issues among the working population.

Concurrently, rapid digitalization in recent years has accelerated societal stress. Numerous studies have linked internet use to mental health outcomes ^(3), (4), (5), (6)^. Considering the rise in internet use and time spent online, assessing the internet’s impact on mental health is essential. Internet gaming disorder (IGD) was included in the Diagnostic and Statistical Manual of Mental Disorders, Fifth Edition, in 2013 as a condition needing further study, and gaming disorder was added to the International Statistical Classification of Diseases and Related Health Problems, 11th Revision, in 2019. As reported by Darvesh et al. ^(7)^, depression is among the most prevalent health issues associated with IGD.

The internet is becoming an integral part of daily life. The Ministry of Internal Affairs and Communications (MIC) in Japan reported in 2024 that the average person spends approximately 3 hours per day online, exceeding television viewing time ^(8)^. Data released by the MIC in 2019 indicated that the average time spent online daily was approximately 2 hours, suggesting a notable increase in internet use over the last 5 years ^(8)^. Among those in their 20s, mean daily internet use is approximately 5 hours. The widespread use of the internet warrants a close look at its potential health impacts.

Various studies have been conducted on the relationship between internet use and depression; however, most have focused on younger populations ^(9)^. In Japan, a study ^(10)^ reported an association between internet addiction and depression among university students, but no such studies have been conducted on the working-age population. Nevertheless, as individuals of various ages increasingly use the internet ^(8)^, assessing its impact across age groups is crucial. Furthermore, given the relationship between depression and various environmental factors ^(11)^, it is essential to evaluate depression in workers, considering work, family, and health contexts. We focused on off-duty internet use because our previous study ^(12)^ suggested that prolonged engagement in such activities is associated with poor mental health, particularly among adolescents.

Therefore, this study aimed to comprehensively analyze the relationship between internet use and depression, particularly examining whether off-duty internet use is independently associated with depression beyond known risk factors.

## Materials and Methods

### Study and questionnaire

The Japanese Civil Servants (JACS) study is an international collaborative study that has been conducted at 5- to 6-year intervals since 1998 in conjunction with the Whitehall II study (British Civil Servants study) and the Helsinki Health Survey. Items in the JACS study were selected from the Whitehall II study and translated into Japanese, then back-translated into English by an individual unacquainted with the original questionnaire. The research team responsible for the Whitehall II study verified the accuracy of this back-translation.

### Participants

This study included local government officials from the JACS study phase 5, conducted in 2019, consisting of civil servants from a prefecture on Japan’s west coast. Of the 4,406 participants in the JACS study phase 5, the survey was completed by 3,751 (85.1%). In our study, we included 3,403 participants (men: 59.6%, women: 40.4%); those not aged 20-65 years and with missing values for the variables used in the analysis were excluded. This study was approved by the Institutional Review Board of the University of Toyama (approval number: R2020019). The participants provided informed consent and voluntarily participated in the study.

### Measures

#### Working environments

Participants’ work environments were evaluated based on their job class, overtime work hours, and the Karasek model of job strain ^(13), (14)^.

Job classes were categorized as low, intermediate, or high, while monthly overtime hours were categorized as <30, 30-45, or ≥45 hours. Monthly overtime hours were defined as the total monthly duration of overtime work on weekdays plus work hours on weekends and holidays.

This study employed a job strain (demands-control-support) model ^(14)^ to assess psychosocial work characteristics, comprising 25 self-reported items on job control (15 items), job demands (four items), and social support (six items) ^(13)^. Response categories ranged from 0 (often) to 3 (never). After recording all items in a consistent direction, the scores for each scale were calculated by summing item scores. Subsequently, participants were classified into tertiles based on their scores. A high score on each scale indicated a high level of control, demands, or support at work. In this study population, Cronbach’s alpha ^(15)^ was 0.78, 0.66, and 0.88 for control, demands, and support at work, respectively.

#### Household characteristics

The characteristics of the participants’ households were assessed using the following variables: marital status, caregiving, and work-family conflict. Marital status was categorized as married or unmarried, and caregiving was assessed based on whether the participant was a primary caregiver.

Conflict between family and work was assessed using four items to measure both family-to-work conflict and work-to-family conflict ^(16)^. The scale assesses how work responsibilities affect family life and vice versa. Items on work-to-family conflict evaluate how work responsibilities impact family life, while family-to-work items assess how family life affects work performance. Responses were recorded on a scale ranging from 0 (never) to 2 (often).

The total score, indicating the degree of conflict, was calculated by summing individual scores, and these totals were classified into tertiles: “low,” “medium,” or “high” ^(17)^. Cronbach’s alpha was 0.83 for family-to-work conflict and 0.71 for work-to-family conflict in this study population.

#### Health risk behaviors and health status

Participants’ health status was evaluated based on several variables, including alcohol consumption, smoking habits, chronic diseases, and sleep quality. Daily alcohol consumption was assessed with two questions: “How much alcohol do you drink at one time?” and “What is the average number of units you drink per day?” Individuals who consumed four or more units daily were classified as heavy drinkers based on the guidelines of the Ministry of Health, Labour and Welfare ^(18)^. In this study, one unit of alcohol was defined as 20 g of alcohol or approximately 180 mL of Japanese sake, 500 mL of beer, or 350 mL of a canned cocktail. Current smoking status was dichotomized into two categories: smokers and nonsmokers. Chronic disease presence was determined by asking each participant whether they currently had a chronic disease. Sleep quality was measured using the Japanese version of the Pittsburgh Sleep Quality Index (PSQI) ^(19), (20)^ with a cutoff score of 6, classifying participants into two groups based on their PSQI scores. Regarding sleep duration, based on our previous research ^(21)^, we divided participants into the following three groups: <6, 6-9, or ≥9 hours.

#### Internet use

The study evaluated electronic device use duration, including internet use at work and private internet use on weekdays. Device use was classified into <4, 4-7, and >7 hours. Similarly, time spent on the internet was categorized as <2, 2-4, and >4 hours, based on the results of our previous studies with elementary school children ^(12)^.

#### Depression

Depression was assessed using the Japanese version of the Center for Epidemiologic Studies Depression Scale (CES-D) ^(22)^, a highly valid and reliable test. The CES-D comprises 20 items, 16 of which are negative and indicate the presence of symptoms of depression, such as depressed mood, physical symptoms, and interpersonal difficulties. The remaining four items are positive and indicate the absence of depression symptoms, such as a positive mood. Participants rated the frequency of symptoms experienced over the preceding week on a scale of 1-4. The scale included the following options: “none” (1 point), “1-2 days” (2 points), “3-4 days” (3 points), or “≥5 days” (4 points). In this study, scores of ≥19 and ≤18 indicate the presence and absence of depression, respectively. The cutoff scores were derived from a study conducted on Japanese workers ^(23)^. Furthermore, the analysis included sex and age as variables.

### Statistical analysis

First, statistical models were constructed separately for men and women to examine the factors associated with depression. Next, a combined model was analyzed to explore factors related to sex differences. A chi-square test was used to evaluate the potential for sex-based differences in all variables used in this study. Logistic regression analyses examined whether depression could be explained by internet use, work, household, and health characteristics, with odds ratios (ORs) and 95% confidence intervals (95% CIs) calculated. Statistical analysis was conducted using SPSS version 23.0 (IBM Corp). p-Values <0.05 were considered statistically significant.

## Results

The final analysis included 3,403 participants (Table 1). The results revealed a significant sex difference in depression prevalence: 31.4% of women experienced depression, compared to 26.1% of men. No significant sex differences were found in the time spent on the internet for personal use. In the work environment, men were more likely to work longer overtime hours, while women exhibited lower job control, demands, and support levels. Work-family conflict was also more prevalent among women. Regarding health variables, men were more likely to be heavy drinkers and smokers and have chronic diseases, whereas women were more likely to experience impaired sleep quality.

**Table 1.** Participants’ Characteristics (n = 3,751).

Characteristics		Men (n = 2,227), n (%)	Women (n = 1,492), n (%)	χ^2^ p-Value
Depression				<0.001
	No	1,645 (73.9)	1,023 (68.6)	
	Yes	582 (26.1)	469 (31.4)	
Age (years)				<0.001
	20-29	345 (15.5)	466 (31.2)	
	30-39	333 (15.0)	337 (22.6)	
	40-49	608 (27.3)	376 (25.2)	
	≥50	941 (42.3)	313 (21.0)	
Electronic device use at work (hours per day)				<0.001
	<4	677 (30.4)	532 (35.7)	
	4-7	1,093 (49.1)	600 (40.2)	
	≥7	457 (20.5)	360 (24.1)	
Internet use in private life (hours per day)				0.530
	<2	1,325 (59.7)	892 (59.9)	
	2-4	754 (34.0)	489 (32.9)	
	≥4	141 (6.4)	107 (7.2)	
Job class				<0.001
	Low	1,286 (58.0)	1,218 (81.9)	
	Intermediate	512 (23.1)	195 (13.1)	
	High	421 (19.0)	75 (5.0)	
Overtime (hours per month)				<0.001
	<30	1,698 (76.2)	1,195 (80.1)	
	30-45	337 (15.1)	226 (15.1)	
	≥45	192 (8.6)	71 (4.8)	
Job control				0.004
	High	738 (33.1)	449 (30.1)	
	Intermediate	748 (33.6)	468 (31.4)	
	Low	741 (33.3)	575 (38.5)
Job demands				<0.001
	Low	1,116 (50.1)	577 (38.7)	
	Intermediate	657 (29.5)	488 (32.7)
	High	454 (20.4)	427 (28.6)
Support at work				<0.001
	High	584 (26.2)	505 (33.8)	
	Intermediate	822 (36.9)	543 (36.4)	
	Low	821 (36.9)	444 (29.8)	
Married				<0.001
	Yes	1,712 (77.0)	834 (56.0)	
	No	511 (23.0)	654 (44.0)	
Caregiving				0.372
	No	1,781 (80.6)	1,175 (79.4)	
	Yes	429 (19.4)	305 (20.6)	
Work-to-family conflict				<0.001
	Low	757 (34.3)	331 (22.4)	
	Intermediate	765 (34.6)	525 (35.5)	
	High	687 (31.1)	621 (42.0)	
Family-to-work conflict				0.002
	Low	1,040 (47.3)	652 (44.2)	
	Intermediate	588 (26.7)	362 (24.5)	
	High	571 (26.0)	462 (31.3)	
Heavy drinking				<0.001
	No	1,956 (92.9)	1,369 (95.6)	
	Yes	149 (7.1)	63 (4.4)	
Smoking				<0.001
	No	1,835 (83.0)	1,464 (98.8)	
	Yes	375 (17.0)	18 (1.2)	
Longstanding illness				<0.001
	No	1,408 (64.0)	1,146 (77.6)	
	Yes	791 (36.0)	331 (22.4)	
Poor sleep quality				<0.001
	No	1,543 (69.3)	951 (63.7)	
	Yes	684 (30.7)	541 (36.3)	
Sleep duration (hours per day)			
	<6	487 (22.0)	363 (24.5)	0.205
	6-9	1,705 (77.1)	1,104 (74.6)	
	≥9	19 (0.9)	12 (0.8)	

Notes: Pearson’s chi-square tests were used to examine differences in categorical participant characteristics between men and women.

Table 2 presents the univariate ORs for depression for each variable. The OR for women (1.30, 95% CI 1.12-1.50) was statistically significant. Electronic device use for >7 hours per day was associated with depression. Moreover, internet use for personal reasons was linked to depression when used for >2 hours per day, with a stronger correlation observed when used for >4 hours. Correlations were observed between depression and variables pertaining to both the work environment and the household. Except for smoking, all health-related variables were associated with depression.

**Table 2.** Prevalence of Depressive Symptoms and Univariate Logistic Regression Analysis of Factors Associated with Depression.

Characteristics		Prevalence of depressive symptoms (%)	OR (95% CI)
Sex: female		31.4	**1.30** (1.12-1.50)
Age (years)			
	20-29	29.2	Ref
	30-39	29.2	1.00 (0.80-1.25)
	40-49	29.7	1.02 (0.84-1.25)
	≥50	25.6	0.83 (0.69-1.01)
Electronic device use at work (hours per day)			
	<4	26.5	Ref
	4-7	26.8	1.02 (0.86-1.20)
	≥7	33.4	**1.39** (1.15-1.69)
Internet use in private life (hours per day)			
	<2	26.2	Ref
	2-4	29.8	**1.20** (1.03-1.40)
	≥4	35.5	**1.55** (1.18-2.05)
Job class			
	Low	30.3	Ref
	Intermediate	27.6	0.88 (0.73-1.06)
	High	18.8	**0.53** (0.42-0.68)
Overtime (hours per month)			
	<30	26.1	Ref
	30-45	34.9	**1.51** (1.25-1.83)
	≥45	35.7	**1.57** (1.20-2.05)
Job control			
	High	17.2	Ref
	Intermediate	26.7	**1.75** (1.43-2.13)
	Low	39.2	**3.10** (2.57-3.73)
Job demands			
	Low	20.5	Ref
	Intermediate	29.4	**1.61** (1.35-1.91)
	High	41.3	**2.72** (2.28-3.25)
Support at work			
	High	18.0	Ref
	Intermediate	25.8	**1.58** (1.30-1.92)
	Low	39.3	**2.94** (2.43-3.56)
Married			
	No	33.5	**1.45** (1.25-1.69)
Caregiving			
	Yes	33.9	**1.39** (1.17-1.66)
Work-to-family conflict			
	Low	10.6	Ref
	Intermediate	23.0	**2.52** (2.00-3.18)
	High	48.6	**7.96** (6.39-9.93)
Family-to-work conflict			
	Low	18.0	Ref
	Intermediate	25.3	**1.54** (1.27-1.86)
	High	48.3	**4.25** (3.57-5.05)
Heavy drinking			
	Yes	34.9	**1.40** (1.05-1.88)
Smoking			
	Yes	27.3	0.94 (0.75-1.19)
Longstanding illness
	Yes	33.3	**1.40** (1.20-1.62)
Poor sleep quality			
	Yes	53.2	**6.01** (5.15-7.02)
Sleep duration (hours per day)			
	<6	39.2	**1.93** (1.64-2.27)
	6-9	25.0	Ref
	≥9	22.6	0.87 (0.38-2.04)

Notes: Correlations were evaluated using univariable logistic regressions and expressed as ORs with 95% CIs. CI: confidence interval; OR: odds ratio. Statistically significant values (p < 0.05) are shown in bold.

Tables 3 and 4 present the results of the multivariate analysis of depression-related factors by sex. Model 1 included sex, age, internet use, and work environment variables. Model 2 additionally included variables related to the household environment. Model 3 additionally included health-related variables. Univariate analysis indicated a correlation between the duration of exposure to electronic devices at work and depressive symptom prevalence. However, this association became nonsignificant when work environment factors were considered. For both sexes, >4 hours of daily internet use for personal reasons was associated with depression. Depression prevalence was lower among those in high job classes; however, this trend was observed exclusively among men. Moderate overtime was associated with lower levels of depression only in men. In Model 3, no association was observed between job demands and depression among women. In both sexes, there was an association between work-family conflict, marital status, poor sleep quality, and depression. Furthermore, chronic illness was identified as a significant predictor of depression exclusively among men. Regarding sleep duration, <6 hours was associated with fewer depressive symptoms in men only.

**Table 3.** Multivariable Logistic Regression Analysis of Factors Associated with Depression in Men.

Characteristics		Model 1	Model 2	Model 3
		OR (95% CI)	OR (95% CI)	OR (95% CI)
Age (years)				
	20-29	Ref	Ref	Ref
	30-39	0.84 (0.58-1.22)	0.73 (0.48-1.11)	0.72 (0.46-1.14)
	40-49	1.03 (0.73-1.46)	1.02 (0.69-1.50)	0.85 (0.56-1.31)
	≥50	1.09 (0.76-1.56)	1.20 (0.80-1.80)	0.87 (0.55-1.38)
Electronic device use at work (hours per day)				
	<4	Ref	Ref	Ref
	4-7	1.15 (0.90-1.48)	1.10 (0.84-1.45)	1.12 (0.83-1.50)
	≥7	1.08 (0.79-1.47)	1.01 (0.72-1.41)	1.04 (0.72-1.51)
Internet use in private life (hours per day)				
	<2	Ref	Ref	Ref
	2-4	**1.26** (1.01-1.57)	**1.36** (1.07-1.74)	1.28 (0.98-1.68)
	≥4	**1.62** (1.06-2.49)	**1.89** (1.17-3.08)	**1.83** (1.08-3.10)
Job class				
	Low	Ref	Ref	Ref
	Intermediate	0.87 (0.66-1.15)	0.84 (0.62-1.14)	0.86 (0.62-1.19)
	High	**0.60** (0.42-0.84)	**0.58** (0.40-0.84)	**0.61** (0.41-0.90)
Overtime (hours per month)				
	<30	Ref	Ref	Ref
	30-45	0.95 (0.71-1.28)	0.73 (0.53-1.00)	**0.70** (0.49-0.99)
	≥45	0.99 (0.67-1.45)	0.81 (0.54-1.23)	0.85 (0.55-1.33)
Job control				
	High	Ref	Ref	Ref
	Intermediate	**1.72** (1.31-2.27)	**1.66** (1.23-2.23)	**1.87** (1.35-2.59)
	Low	**2.89** (2.19-3.80)	**2.82** (2.09-3.80)	**2.93** (2.11-4.07)
Job demands				
	Low	Ref	Ref	Ref
	Intermediate	**2.07** (1.62-2.65)	**1.37** (1.04-1.79)	**1.52** (1.13-2.04)
	High	**3.45** (2.59-4.59)	**1.55** (1.12-2.16)	**1.67** (1.17-2.39)
Support at work				
	High	Ref	Ref	Ref
	Intermediate	**1.39** (1.05-1.86)	1.22 (0.89-1.65)	1.30 (0.93-1.82)
	Low	**2.34** (1.76-3.12)	**1.88** (1.38-2.56)	**1.92** (1.37-2.69)
Married				
	No	NA	**1.74** (1.29-2.34)	**1.52** (1.10-2.09)
Caregiving				
	Yes	NA	1.07 (0.80-1.43)	1.02 (0.75-1.40)
Work-to-family conflict				
	Low	NA	Ref	Ref
	Intermediate	NA	**2.33** (1.68-3.23)	**2.10** (1.48-2.99)
	High	NA	**5.97** (4.15-8.59)	**4.67** (3.14-6.94)
Family-to-work conflict				
	Low	NA	Ref	Ref
	Intermediate	NA	**1.36** (1.01-1.82)	1.32 (0.96-1.81)
	High	NA	**2.82** (2.10-3.80)	**2.49** (1.80-3.44)
Heavy drinking				
	Yes	NA	NA	0.96 (0.60-1.53)
Smoking				
	Yes	NA	NA	0.99 (0.72-1.36)
Longstanding illness				
	Yes	NA	NA	**1.35** (1.03-1.76)
Poor sleep quality				
	Yes	NA	NA	**4.58** (3.51-5.97)
Sleep duration (hours per day)				
	<6	NA	NA	**0.71** (0.52-0.95)
	6-9	NA	NA	1.00
	≥9	NA	NA	1.38 (0.37-5.21)

Notes: Correlations were evaluated simultaneously using multiple logistic regressions and expressed as ORs with 95% CIs; Model 1 is adjusted for age, internet use, and working environments; Model 2 is adjusted for age, internet use, working environments, and household characteristics (marital status, primary caregiver status, and work-family conflict); Model 3 is adjusted for age, internet use, working environments, household characteristics, and health risk behaviors and health status. CI: confidence interval; NA: not applicable; OR: odds ratio. Statistically significant values (p < 0.05) are shown in bold.

**Table 4.** Multivariable Logistic Regression Analysis of Factors Associated with Depression in Women.

Characteristics		Model 1	Model 2	Model 3
		OR (95% CI)	OR (95% CI)	OR (95% CI)
Age (years)				
	20-29	Ref	Ref	Ref
	30-39	1.12 (0.80-1.56)	1.00 (0.69-1.46)	0.97 (0.64-1.47)
	40-49	1.00 (0.70-1.42)	0.81 (0.55-1.21)	0.82 (0.53-1.28)
	≥50	0.91 (0.60-1.40)	0.85 (0.53-1.39)	0.71 (0.41-1.23)
Electronic device use at work (hours per day)				
	<4	Ref	Ref	Ref
	4-7	0.83 (0.63-1.10)	0.75 (0.56-1.01)	0.85 (0.61-1.17)
	≥7	1.10 (0.80-1.50)	1.14 (0.82-1.61)	1.27 (0.88-1.83)
Internet use in private life (hours per day)				
	<2	Ref	Ref	Ref
	2-4	**1.36** (1.03-1.81)	**1.44** (1.05-1.98)	1.21 (0.86-1.71)
	≥4	**2.27** (1.43-3.63)	**2.71** (1.65-4.47)	**2.31** (1.32-4.05)
Job class				
	Low	Ref	Ref	Ref
	Intermediate	0.99 (0.67-1.48)	0.93 (0.61-1.43)	0.87 (0.55-1.37)
	High	0.93 (0.49-1.78)	0.89 (0.44-1.79)	0.80 (0.37-1.70)
Overtime (hours per month)				
	<30	Ref	Ref	Ref
	30-45	**1.53** (1.11-2.11)	1.12 (0.79-1.59)	1.18 (0.80-1.73)
	≥45	1.68 (0.97-2.88)	1.20 (0.67-2.15)	1.23 (0.66-2.29)
Job control				
	High	Ref	Ref	Ref
	Intermediate	**1.42** (1.03-1.95)	1.29 (0.92-1.82)	1.16 (0.80-1.69)
	Low	**2.12** (1.55-2.92)	**2.04** (1.45-2.87)	**1.74** (1.21-2.52)
Job demands				
	Low	Ref	Ref	Ref
	Intermediate	**1.43** (1.07-1.92)	1.08 (0.78-1.49)	0.97 (0.69-1.38)
	High	**2.11** (1.57-2.85)	**1.42** (1.02-1.98)	1.27 (0.88-1.82)
Support at work				
	High	Ref	Ref	Ref
	Intermediate	**1.63** (1.21-2.20)	**1.45** (1.05-2.01)	**1.43** (1.01-2.02)
	Low	**3.08** (2.25-4.23)	**2.48** (1.77-3.49)	**2.25** (1.56-3.25)
Married				
	No	NA	**2.03** (1.49-2.76)	**1.77** (1.26-2.48)
Caregiving				
	Yes	NA	1.16 (0.85-1.59)	1.23 (0.87-1.75)
Work-to-family conflict				
	Low	NA	Ref	Ref
	Intermediate	NA	**1.90** (1.24-2.90)	1.49 (0.95-2.35)
	High	NA	**4.84** (3.12-7.51)	**3.61** (2.25-5.77)
Family-to-work conflict				
	Low	NA	Ref	Ref
	Intermediate	NA	**1.43** (1.01-2.03)	1.44 (0.99-2.10)
	High	NA	**3.00** (2.13-4.23)	**2.74** (1.89-3.99)
Heavy drinking				
	Yes	NA	NA	1.46 (0.77-2.78)
Smoking				
	Yes	NA	NA	0.67 (0.21-2.15)
Longstanding illness				
	Yes	NA	NA	0.92 (0.65-1.29)
Poor sleep quality				
	Yes	NA	NA	**4.19** (3.11-5.66)
Sleep duration (hours per day)				
	<6	NA	NA	0.80 (0.57-1.12)
	6-9	NA	NA	1.00
	≥9	NA	NA	0.32 (0.04-2.75)

Notes: Correlations were evaluated simultaneously using multiple logistic regressions and expressed as ORs with 95% CIs; Model 1 is adjusted for age, internet use, and working environments; Model 2 is adjusted for age, internet use, working environments, and household characteristics (marital status, primary caregiver status, and work-family conflict); Model 3 is adjusted for age, internet use, working environments, household characteristics, and health risk behaviors and health status. CI: confidence interval; NA: not applicable; OR: odds ratio. Statistically significant values (p < 0.05) are shown in bold.

Table 5 presents the results of the multivariate analysis for both sexes. The OR for women (1.19, 95% CI 1.01-1.40) in Model 1 was statistically significant. However, after the introduction of variables related to the household environment in Model 2, the OR decreased and was no longer statistically significant. Internet use for >2 hours per day showed a dose-response relationship with depressive symptoms. Variables related to the work environment, except for overtime hours, were linked to depression. Furthermore, work-family conflict, particularly work disruptions to family life, showed a significant association with depression (OR 4.18, 95% CI 3.10-5.63) in the fully adjusted model. Among health variables, poor sleep quality and sleep duration were associated with depression.

**Table 5.** Multivariable Logistic Regression Analysis of Factors Associated with Depression in All Participants.

Characteristics		Model 1	Model 2	Model 3
		OR (95% CI)	OR (95% CI)	OR (95% CI)
Sex: female		**1.19** (1.01-1.40)	0.89 (0.74-1.07)	0.86 (0.70-1.05)
Age (years)				
	20-29	Ref	Ref	Ref
	30-39	0.96 (0.75-1.22)	0.85 (0.64-1.12)	0.81 (0.60-1.10)
	40-49	1.00 (0.79-1.28)	0.90 (0.69-1.18)	0.82 (0.61-1.11)
	≥50	1.02 (0.78-1.33)	1.04 (0.77-1.41)	0.83 (0.59-1.16)
Electronic device use at work (hours per day)				
	<4	Ref	Ref	Ref
	4-7	1.00 (0.84-1.21)	0.92 (0.76-1.13)	0.97 (0.78-1.20)
	≥7	1.10 (0.88-1.36)	1.06 (0.83-1.34)	1.13 (0.87-1.46)
Internet use in private life (hours per day)				
	<2	Ref	Ref	Ref
	2-4	**1.30** (1.09-1.55)	**1.40** (1.16-1.70)	**1.25** (1.01-1.54)
	≥4	**1.86** (1.36-2.54)	**2.20** (1.56-3.10)	**1.98** (1.36-2.88)
Job class				
	Low	Ref	Ref	Ref
	Intermediate	0.94 (0.75-1.18)	0.92 (0.72-1.17)	0.88 (0.68-1.15)
	High	**0.68** (0.51-0.92)	**0.68** (0.49-0.93)	**0.66** (0.47-0.93)
Overtime (hours per month)				
	<30	Ref	Ref	Ref
	30-45	1.18 (0.95-1.47)	0.88 (0.70-1.12)	0.90 (0.70-1.16)
	≥45	1.20 (0.88-1.63)	0.89 (0.64-1.24)	0.93 (0.65-1.33)
Job control				
	High	Ref	Ref	Ref
	Intermediate	**1.59** (1.29-1.95)	**1.50** (1.20-1.88)	**1.51** (1.18-1.91)
	Low	**2.53** (2.06-3.11)	**2.44** (1.96-3.05)	**2.29** (1.80-2.92)
Job demands				
	Low	Ref	Ref	Ref
	Intermediate	**1.76** (1.46-2.12)	1.23 (1.00-1.51)	1.22 (0.98-1.53)
	High	**2.75** (2.24-3.37)	**1.52** (1.20-1.91)	**1.48** (1.15-1.90)
Support at work				
	High	Ref	Ref	Ref
	Intermediate	**1.49** (1.21-1.83)	**1.34** (1.07-1.67)	**1.37** (1.08-1.74)
	Low	**2.66** (2.16-3.29)	**2.20** (1.75-2.75)	**2.12** (1.66-2.70)
Married				
	No	NA	**1.92** (1.55-2.37)	**1.67** (1.33-2.10)
Caregiving				
	Yes	NA	1.11 (0.90-1.37)	1.11 (0.88-1.40)
Work-to-family conflict				
	Low	NA	Ref	Ref
	Intermediate	NA	**2.17** (1.68-2.81)	**1.86** (1.41-2.45)
	High	NA	**5.40** (4.09-7.12)	**4.18** (3.10-5.63)
Family-to-work conflict				
	Low	NA	Ref	Ref
	Intermediate	NA	**1.37** (1.10-1.72)	**1.39** (1.09-1.76)
	High	NA	**2.83** (2.27-3.53)	**2.60** (2.04-3.31)
Heavy drinking				
	Yes	NA	NA	1.14 (0.79-1.64)
Smoking				
	Yes	NA	NA	0.98 (0.73-1.33)
Longstanding illness				
	Yes	NA	NA	1.16 (0.94-1.43)
Poor sleep quality				
	Yes	NA	NA	**4.27** (3.51-5.19)
Sleep duration (hours per day)				
	<6	NA	NA	**0.75** (0.61-0.94)
	6-9	NA	NA	1.00
	≥9	NA	NA	0.81 (0.28-2.39)

Notes: Correlations were evaluated simultaneously using multiple logistic regressions and expressed as ORs with 95% CIs; Model 1 is adjusted for age, internet use, and working environments; Model 2 is adjusted for age, internet use, working environments, and household characteristics (marital status, primary caregiver status, and work-family conflict); Model 3 is adjusted for age, internet use, working environments, household characteristics, and health risk behaviors and health status. CI: confidence interval; NA: not applicable; OR: odds ratio. Statistically significant values (p < 0.05) are shown in bold.

## Discussion

This study demonstrated that, along with stressful work and family conditions and health status, prolonged off-duty internet use was independently associated with depression among Japanese civil servants. We found no statistically significant association between electronic device use at work and depression. This contrast supports our interpretation that the adverse effects are likely driven by the nature or content of the use rather than the screen time itself. Additionally, low job class, overtime, high demands, chronic illness, and sleep duration were associated with depression only among men, whereas no associated factors were found exclusively in women. Regarding sex differences in depression, associations between sex and depression were no longer observed when household-related variables were introduced into the model.

These findings are consistent with those of previous studies. Lin et al. ^(9)^ reported a dose-response relationship between social network use and depression among young adults in the United States. Similarly, Boers et al. ^(24)^ observed an association between increased computer use and depressive symptoms among adolescents. While most previous studies have focused on young people, including students, this study found similar results for working people after adjusting for various workplace- and life-related variables. Internet use through social networking sites (SNSs) may precipitate the onset of depression. Primack et al. ^(6)^ examined the association between SNSs and depression and reported that depression increased with increased time spent on SNSs. However, baseline depression was not associated with increased social media use at follow-up. A meta-analysis by Saiphoo et al. ^(25)^ showed that SNS use was associated with lower self-esteem. Murthy ^(26)^ noted that checking social media can involve comparing one’s everyday life to others’ highlights, potentially reducing self-esteem. Alienation and life dissatisfaction may increase when SNS posts themselves—rather than fostering connections—become the focus.

Moreover, internet use for communication may not facilitate the formation of human connections. Online shopping, for example, enables people to shop at any time and from any location. However, Murthy ^(26)^ suggests that in exchange for the internet’s efficiency, people are increasingly giving up real human interaction in their daily lives. Turkle ^(27)^ notes that virtual connections may risk objectifying others and that these connections offer only uncertain commitments, potentially leading to isolation. Thus, despite prevalent internet use, users may still experience difficulties forming connections and developing relationships, contributing to feelings of emptiness and, in some cases, depressive symptoms. Furthermore, prolonged internet use may lead to depression due to information overload. Matthes et al. ^(5)^ linked perceived information overload to depression, citing that it can lead to psychological stress, exhaustion, and anxiety. Information overload is associated with the use of SNSs and reportedly affects all age groups, not only young people.

The aforementioned possible negative effects of internet use may intensify with prolonged exposure, which can adversely affect mental health. The observed association between prolonged off-duty internet use and depression may be mediated by the mechanisms discussed earlier, including superficial interpersonal connections and information overload.

In addition to internet use for personal purposes, job control and support, marital status, and work-family conflict were found to be associated with depression in both men and women. These findings were consistent with those of our previous study. An earlier study demonstrated that low job control impedes recovery from depression ^(28)^, while another study highlighted the link between low job support and poor mental health ^(17)^. Regarding marital status, a previous study demonstrated that being unmarried was associated with depression onset ^(28)^. Our study also supports this finding. The relationship between work-family conflict and mental health is also consistent with our previous studies’ findings. Sekine et al. ^(29)^ demonstrated that work-family conflict is associated with a decline in mental health, with the relationship being particularly pronounced for work-to-family conflict.

In this study, lower job class, chronic illness, overtime, sleep duration, and high job demands were associated with depression only among men. A study that analyzed factors related to mental health by sex demonstrated that job class and chronic illness were associated exclusively in men ^(29)^, consistent with our study’s results. The reason for the observed association between moderate overtime, shorter sleep duration, and decreased depression in men is unclear. One possible explanation is that moderate overtime may be feasible only for those with good mental health, while workers with poorer mental health may have reduced workloads at the company’s discretion. Among those with the greatest amount of overtime, the association may not be significant, as the group includes both individuals capable of overtime due to good mental health and those experiencing depressive symptoms from excessive overtime. Among men, a tendency toward fewer depressive symptoms was observed in the group with shorter sleep duration. The absence of a similar association in women remains unclear, and further investigation is warranted. Although the reason for the lack of an association between job demands and depression in women in the fully adjusted model is unclear, this study suggests that work-family conflict may be more strongly associated with job demands in women and that high job demands may be more strongly associated with poor sleep quality.

As shown in Table 5, the analysis of all participants revealed a decrease in the OR and a lack of statistical significance for sex variables in the model adjusted for family variables. This may reflect the disappearance of sex differences in depression upon adjusting for work-family conflict. As illustrated in Table 1, the proportion of women experiencing work-family conflict was higher, likely contributing to the elevated depression prevalence among women. This adjustment in Model 2 may have rendered the sex variable nonsignificant. Puthran et al. ^(30)^ reported no statistically significant difference in depression prevalence between male and female medical students. In contrast, a study of US medical residents reported that the increase in depression scores 6 months after the start of internship training was higher for women than for men ^(31)^. However, this difference decreased after adjusting for work-family conflict, consistent with our study’s results. Additionally, a previous study indicated that sex differences in poor mental functioning were attenuated when work-family conflict was considered ^(29)^. Similarly, a previous study using comparable variables reported that sex disparities existed in regard to poor mental health, fatigue, and poor sleep quality in a model that was unadjusted for work-family conflict ^(32)^. These findings imply that work-family conflict exposes sex differences in depression.

This study has some limitations. First, as this was a cross-sectional study, causal relationships cannot be established. Prior research indicates that individuals with depression may engage in increased internet use to access health information and suicide-related content ^(33), (34)^. Therefore, longitudinal studies are needed to clarify the relationship between internet use and depressive symptoms. However, as mentioned previously, internet use is highly likely to trigger depression.

Second, although this study analyzed time spent online, it did not account for specific content accessed. Internet use encompasses diverse activities, such as email communication, web browsing, gaming, and SNS use. For example, it has been demonstrated that exposure to violent content or pornography on the internet can negatively impact mental health ^(4)^. Conversely, it has been shown that using SNSs to connect with others can reduce feelings of loneliness ^(35)^. Therefore, it is presumed that the impact on mental health depends on the specific content and purposes of internet use. Further research must include content and purpose analyses.

Third, the purpose and patterns of internet use may differ between the time this study’s survey was conducted and the present. A comparison of reports from the MIC in 2020 and 2024 ^(8)^ indicates that time spent on social media has increased. Furthermore, the use of video-sharing and content-posting platforms has grown substantially. Given these trends, our findings, which are based on earlier data, may underestimate the strength of the association between off-duty internet use and depression in the current context. Therefore, the magnitude of this issue may be greater at present than suggested by our results.

Finally, because the study population consisted exclusively of civil servants, the representativeness of the sample may be limited. Data from the Ministry of Education, Culture, Sports, Science and Technology ^(36)^ and the MIC ^(37)^ indicate that civil servants include a higher proportion of university graduates than the general population. Civil servants typically have higher educational attainment and incomes, contributing to a higher-than-average socioeconomic status. As factors influencing depression may vary across income groups, the generalizability of the findings may be constrained.

## Conclusions

This study’s findings suggest that, in addition to work stress and family characteristics, off-duty internet use is independently associated with depressive symptoms among Japanese civil servants, and the strength of the association demonstrated a positive correlation with the duration of use. These results highlight the necessity for further research into the potential impact of internet use on mental health, particularly given the rising trends in online activity. However, to validate this study’s findings, longitudinal studies analyzing the content and purpose of internet use across a more diverse study population are needed.

## Article Information

### Author Contributions

Daiki Fukushima designed the study, developed the main conceptual ideas, collected the data, and prepared the proof outline. Takashi Tatsuse, Michikazu Sekine, and Masaaki Yamada aided in interpreting the results and contributed to the manuscript. Michikazu Sekine supervised the project. Daiki Fukushima wrote the manuscript with support from Takashi Tatsuse, Michikazu Sekine, and Masaaki Yamada. All authors discussed the results and commented on the manuscript.

### Conflicts of Interest

None

### Ethics Approval

This study was approved by the Institutional Review Board of the University of Toyama (approval number: R2020019).

### Participant Consent

The participants provided informed consent and voluntarily participated in the study.

### Data Availability

The data are available upon reasonable request.
